# Reconciling a Broken Heritage: Developing Mental Health Social Work in Guyana

**DOI:** 10.3390/ijerph20206931

**Published:** 2023-10-17

**Authors:** Coya Halley, Stephen Cowden

**Affiliations:** 1 Department of Social Work, Faculty of Social Sciences, University of Guyana, Georgetown P.O. Box 10-1110, Guyana; halleyc@uni.coventry.ac.uk; 2 School of Health and Social Care, Department of Social Work, University of Gloucestershire, Gloucester GL2 9H, UK

**Keywords:** Guyana, mental health, social work, colonialism, trauma

## Abstract

Guyana’s colonial past has left a trail of economic instability, racial polarization, and physical and mental trauma. Despite the progress made since Guyana’s independence in 1966, the remnants of this colonial past continue to shape present-day Guyana. As a result, violence and trauma continue to impact the mental health of the population. This is manifest in endemic problems of domestic violence and racialized social divisions which have created the conditions for rates of suicide which are amongst the highest in the world. The formal mental health provision which exists in Guyana is based primarily on an individualized and largely biomedical model of care. Despite valuable attempts to develop this provision, the difficulty of physically accessing this for some people and the stigma which surround this means that the capacity of this system to address the serious problems which exist is limited. It is also the case that in times of emotional and psychic distress, and in the context of Guyana being a very religious country, many people turn to traditional supernatural healers and remedies for support. In this paper, we discuss what is known as “Obeah”, noting that while this is widely practiced, it remains something of a taboo subject in Guyana. We consider the reasons why these practices and beliefs continue to be influential. However, what neither these biomedical or supernatural perceptions of mental health are able to address is the sociogenic nature of Guyana’s mental health issues, which we argue emerges out of the historic trauma of Guyana’s experience of colonialism and the violence which it engendered. We argue that profound forms of mental distress which exist in Guyana call for an integrative and holistic practice model that contextualizes these problems through a sociogenic lens. Social workers, working collaboratively with other health-related professions, can occupy a critical role in integrating these different conceptions through developing a rights-based model of mental health where the causes of mental ill-health are understood as socially determined.

## 1. Introduction

In 2014, Guyana made the international news headlines on the basis of an invidious statistic; the World Health Organization reported the country as having the highest per capita suicide rate in the world [1]. The Guyanese government responded with a number of important initiatives focused on a National Suicide Prevention Plan (NSPP). Halliwell has described this as based on a “sustained, collaborative and multi-pronged approach”, which acknowledged both “biological and environmental factors” [2]. Initiatives developed have included a national Public Education campaign based on the theme of “Choose Life–Say No to Suicide”, increased funding for the training of primary health care staff in mental health management, and the implementation of a suicide prevention help lines. NGOs such as the Guyana Foundation have also responded through the development of community-based centers in regions with a high incidence of suicide “offering holistic wellness programs to empower individuals to adopt positive lifestyle-related behaviors and practices” [3]. Yet an assessment of these initiatives in 2022 has noted that suicide rates have remained “stubbornly high”; in spite of both government and grass roots efforts at suicide prevention “spanning various projects and investment of considerable resources, the suicide rate has not substantially improved in recent times. In 2019, Guyana [continued to have the] second highest suicide rate in the world” [4] (p. 2). Faced with the scale of this problem, all of these government and NGO initiatives, which have increased funding, the training of specialist staff, and the development of new facilities, are crucial steps forward. At the same, the fact that these initiatives have not brought about enough of the changes so desperately needed means that there is also a need to ask different questions about the nature of this problem. In this discussion, we argue that it is crucial to broaden the frame of reference to how we are thinking about the problem of suicide, and mental health problems more broadly, in Guyana. The central contribution to knowledge and understanding we are making in this paper is that the mental health crisis, which is manifest in this extremely high incidence of suicide, needs to be understood *sociogenically*; that is, we need to understand the way in which the conditions for this are socially produced. Historically, suicide has been seen as both immoral and as criminal. While current initiatives have gone beyond this, mental health issues in Guyana continue to be conceptualized at the level of individual distress. By seeing the issues at the level of the individual, the problem becomes conceptually and practically separated from the wider patterns of violence and structural inequalities which are prevalent throughout Guyana. The sociogenic approach we are calling for does not see suicide simply as a failure of rationality. Instead, we need to shift the focus away from the nature of the individual who is experiencing these severe feelings of hopelessness and despair toward a consideration of the social and relational factors which are creating the conditions for these widely experienced subjective feelings.

As well as understanding the way in which mental health issues are socially produced in contemporary society, a sociogenic approach allows us to situate our understanding of mental distress in Guyana as a problem with historic dimensions. Guyana’s history is that of a colonial plantation society based on the ruthless exploitation of both slaves and indentured laborers. As Clarke has noted, the arrival of Europeans saw “Amerindian populations largely destroyed by colonial contact and exploitation… [as] the social order was reconstructed according to the colonists’ views of the natural order of racial stratification based upon white superiority and black slavery” [5] (p. 491). The very creation of the modern state of Guyana represented a society fundamentally rooted in relationships based on multiple forms of violence and coercion. There is no question that the diverse groups of people who made up the former colony of “British Guiana” looked forward to a new chapter in their history when they were granted “Home Rule” from the British colonists in 1953, and then independence in 1966. The motto of the post-colonial Guyanese state is “One People, One Nation, One Destiny”, and there has been significant progress since the ending of colonialism in terms of economic development and the attempt to build a multi-ethnic nation. However, the departure of the former colonizers does not in itself create the conditions for new and different sets of social relations to develop. The ongoing ethnic polarization between the “Afro-Guyanese” and “East Indians” [6,7] is one of the most prominent examples of this. Guyana’s politics have for some years been characterized by racialized divide known as “apan jhaat”, Guyanese Hindustani for “vote for your own kind”. Leo Despres has described Guyana’s political development in the post-independence period as one where the country’s political parties became “symbols of racial expression… in its most vicious form; [where] parties and leaders are no longer seen as national coalitions but as exclusive ethic groups fostering a kind of ethnic chauvinism” [8] (p. 173). This illustrates the way in which colonial policies which consciously sought to divide different groups of plantation workers on ethnic lines have continued into the present. At the level of social institutions (Joan Mars’ [9] research on the extent of lethal force used by the police in modern day Guyana offers another example of the persistence of the colonial legacy, which is outside the scope of this discussion but relevant nonetheless. Mars notes that the development of state institutions during the colonial period has shaped the behavior and agency of institutions such as the police in the post-colonial period. She argues that the colonial state developed in Guyana with the primary objective of the “social and political control of a vast number of ethnically and culturally diverse people transplanted from Africa and Asia to facilitate economic profiteering in the plantation colony” [9] (p. xvi). The dehumanizing nature of colonial rule inevitably created resistance, which threatened the plantation based ruling class; hence, state institutions such as the police were developed with the express purpose of forcefully suppressing this. Mars argues that the contemporary problem of the “excessive and deadly” use of lethal force by the police in Guyana today “cannot be addressed without an understanding of the enduring legacy of colonialism and its role in the police function” [9] (p. xiv)), as well as at the level of social identity, relationships shaped during the colonial period persist.

In trying to characterize the nature of this historic legacy, we have seen the work of the renowned Guyanese novelist Wilson Harris as prescient. In a discussion published in 1967, Harris wrote about the difficulty for people in his native Guyana as well as across the Caribbean in coming to terms with their historic legacy of slavery and colonialism at the level of subjective identity in the post-independence period. He asked how it was that people could “begin to reconcile the broken parts of such an enormous heritage, especially when those broken parts appear very often like a grotesque series of adventures, volcanic in precipitate effects as well as vulnerable in its human settlement?” [10] (p. 31). In posing this question, Harris is understanding the construction of subjectivity as historically, socially, and politically constituted. By talking about a “broken legacy”, he is pointing to the need to come to terms with this history of violent subjugation, implying that if this is not addressed, then relationships shaped through the “grotesque adventure” of colonialism will persist. Harris’s concept of a “broken heritage” points to the need to address the way in which the Guyana’s past is shaping its present, and we see this idea as a crucial component within the development of a sociogenic approach to understanding suicide in Guyana.

As well as capturing the historical problem at the level of identity, this concept of the “broken heritage” can also be used to think about the way people in Guyana understand the meanings of mental health and mental illness. What emerges here is a significant disjuncture between the biomedical models used in official mental health provision and the fact that for many Guyanese dealing with emotional and psychic distress, they are most likely to turn to traditional supernatural healers and remedies for support, known as “Obeah”. While this is widely practiced, it remains something of a taboo subject in Guyana. As part of the sociogenic lens, we are arguing that, in understanding issues of mental health in Guyana, it is essential that these approaches are not dismissed; rather, they need to be addressed openly and understood as part of the extant belief systems around mental health in Guyana. We conclude by discussing the unique possibility which social work offers in seeking to integrate these different conceptions through developing a rights-based model of mental health, where the causes of mental ill-health are understood as socially determined.

## 2. Colonialism as a “Broken Heritage”

As a modern state, Guyana was formed through the dispossession of the indigenous people and the introduction of slavery and indentured labor, and the lived realities of this were experiences of sexual abuse, rape, murder, poverty, and deplorable living conditions. Jeffrey Alexander’s work on “cultural trauma” offers a useful way of conceptualizing this. Alexander defines this as a situation where “members of a collective feel they have been subjected to a horrendous event that leaves inedible marks upon their group consciousness, marking their memories forever and changing their future identity in fundamental and irrevocable ways” [11] (p. 1–2). In psychotherapy, trauma is understood as an individual experience; however, Alexander and his co-authors point to the way in which this needs to be understood as a collective experience which frames the identities of whole groups of people. Alexander’s work is useful for pointing out the way in which this historic experience can be powerful but is at the same time repressed, given the pain associated with the trauma. The pioneering work of Frantz Fanon develops this discussion still further in pointing to the psychic dimensions of colonialism. Fanon’s work *Black Skin, White Masks* [12] was groundbreaking for the way he understood that the processes of racialized objectification on which colonialism was based were not just about the political economy of colonialism; they were, at the same time, embedded in the psyche of the colonized:

*If the black man is overcome by to such a degree by the desire to be white, it is because he lives in a society that makes his inferiority complex possible… it is to the extent that society creates his difficulties that he finds himself positioned in a neurotic situation.*

[12] (p. 80)

These ideas were developed further in his later book *The Wretched of the Earth* [13], which was deeply influenced by Fanon’s experience of working as a psychiatrist in the psychiatric hospitals in northern Africa, particularly the hospital at Blida-Joinville in Algeria. As someone working within the psychiatric system, Fanon became intensely aware of the way psychiatry medicalized and individualized the psychic distress of Algerian patients while failing to grasp the way colonialism itself was a direct cause of these people’s psychological distress. He described the colonial world in Algeria as one that was

*…cut in two. The dividing line, the frontiers are shown by barracks and police stations. In the colonies it is the policeman and the soldier who are the official, instituted go-betweens, the spokesmen of the settler and his rule of oppression… It is obvious here that the agents of government speak the language of pure force [bringing] violence into the home and into the mind of the native*.

(p. 38)

For Fanon, colonial violence needed to be understood as simultaneously shaping the physical organization of social space as well as the psychic space. In this Manichean world, the natives were seen as incapable of ethical thought or action, compared to white settlers, who embodied “civilization” and “goodness” at the same time as relentlessly asserting their superiority and the inferiority of the colonized. These social tensions forced the colonized person to live psychically “in a state of permanent tension” [12] (p. 41). David Macey has noted that “Fanon always stresses the sociogenic aspects of symptomatology: symptoms did not, in his view, originate from the person’s unconsciousness or repressed sexual impulse as much as from… the internalisation of social conflicts” [14] (p. 64–65). Hanna Goozee makes a related point when she argues “Fanon’s sociogenic psychiatry, his belief that the traumatic suffering of individuals resulted from their environment and his recognition of the complicity of psychiatry in colonial oppression, necessitated [what Fanon called] a ‘complete calling into question of the colonial situation’” [15] (p. 112). This analysis offers much in understanding the situation in contemporary Guyana, allowing us to see the severe levels of ethnic and racial tension, of violence toward women and girls and of suicide, as rooted in the internalization of the dynamics of the colonial period. While this period has formally ended, the patriarchal and racially polarized social identities and systems which it created live on, both institutionally and in the unconscious. Through this process, the “broken heritage” of colonial violence continues to be played out within contemporary social relations, with profound impact on the mental health and wellbeing of the Guyanese people.

## 3. Gender, Colonialism, and Violence against Women

Goozee notes that despite Fanon’s sociogenic adaptation of the principles of psychiatry to engage in a “highly political theorization of colonialism and violence”, he largely fails to discuss issues of gender within this [15] (p. 117). However, she notes that the same principles on which his argument is based should be extended to include this, a point which strongly concurs with the argument in this paper. In the colonial period in Guyana, it was seen as more economically productive to have plantations worked primarily by men. While women also worked on the plantations, women’s bodies came to be of central importance for slaveholders as a means of replenishing the labor force, particularly in the period nearing the abolishment of slavery and indentureship [7,16]. Women’s child-bearing capacity contributed to a profound sexualization of their bodies, as they became units for “breeding” future generations of slaves [16,17]. Sweeney has argued that through slavery “black women’s bodies positioned them as property to be legally used up not just economically but also sexually and reproductively… Rape was inevitable and inescapable in the slave system; the female slave body was always under threat of sexual violation” [18] (p. 57). Colonial control over the family life of slaves was also driven by this dynamic of controlling women’s procreative role within those families [19].

The internalization of the patterns of violence and control which developed during the colonial period on the basis of gender continue to manifest themselves in contemporary Guyana. In Guyana today, there are estimates that as many as 50% of Guyanese women have experienced intimate partner violence [20]. Miriam Roberts-Hinds, who worked as the head counselor at the Guyana Foundation’s Sunrise Center, notes that she

*…could have been of one of the many suicide stories that pepper local newspapers. She became pregnant when she was only 15 after her first sexual experience with a man, eight years older, who became her husband. And she says he physically abused her. According to Roberts-Hinds, young girls in Guyana learn maxims like, “if he doesn’t hit you, he doesn’t love you” and “better the man you know than the one you don’t”. “What [many women in Guyana] have been introduced to as ‘love’ is what we would term abuse” says Roberts-Hinds. ‘That’s what they think as being normal.*

[21]

The way in which men come to understand “women’s bodies in public spaces… as men’s possessions” which they are entitled to “monitor and control” [22] (p. 12) illustrates Fanon’s insight into the way identities shaped in the violence of the colonial period continue to manifest elements of the dehumanization through which these social relations were developed. Patsy Sutherland’s work on women in contemporary Grenada develops this point further, arguing that “inherited dynamics of sexual possession and subjugation continue to characterize and haunt contemporary relationships”. While this is not driven by same forces of commodification as existed during the time of slavery, the dynamics of possession and control [remain] underpinned by the dominant patriarchal discourse, [and] culminate in an environment where women are once again displaced, and their bodies relegated to mere commodities [23] (p. 44).

The dynamics where women and girls are seen as male possessions connects to mental health and suicide in a number of ways. The following account by a Guyanese East Indian woman, Natasha Houston, points to these:

*In 2013, after enduring seven years of domestic abuse, Houston took their two children and left her husband, Richard Lord. But Lord, a sugar-cane cutter with whom she had eloped when she was only 13, found them in a nearby town. In a deadly and drunken fit of violence, he took the lives of their children—along with Houston’s right arm and most of her left hand. He fled into the woods behind their home. Neighbors found him weeks later, dead from an apparent suicide.*

[21]

Paulette Henry’s research has investigated further instances like this, which she describes as “murder–suicide”; that is, where the murder, invariably of a woman, is followed by the suicide, almost always of a man. Henry notes that these murder–suicides “seem to be inherently linked to emotional relationships in Guyana” [24] (p. 30). She notes also that wider studies across the Caribbean offer further evidence for the way in which these murder-suicides “almost always involved intimate-partner relationship conflict, often in the context of the woman threatening to end or leave the relationship” [24] (p. 30). The research of Roopnarine et al. [25] similarly points to the concurrent existence of high rates of suicide and high levels of intimate partner violence. These patterns of violence and coercion that are commonplace in contemporary Guyana have their roots in the way women were seen during the colonial period, and the research discussed above shows the relationship between this and the incidence of suicide. Alongside “murder–suicides” where the suicides are undertaken by men, it is important to note that the levels of male violence described are themselves causal factors for suicide and suicidal ideation amongst women. The first comprehensive survey conducted in Guyana that explores women’s health and life experiences confirms the adverse effects that violence is having on their mental and emotional wellbeing [26], which leads to suicide and suicidal ideations [27]). The research of Peltzer and Pengpid [28] also offers further evidence, pointing to the way that the greatest proportion of suicidal behavior is present amongst female victims of childhood and adult sexual abuse (p. 408).

## 4. Mental Health and Illness in Post-Colonial Guyana

In the pattern of British colonies throughout the 19th Century, Guyana adopted the Victorian Poor Law approach to welfare service delivery, where recourse is provided as a last resort based on strict eligibility criteria [29,30]. The premier hospital that offers mental health support was formerly known as the “Lunatic Asylum” and was built in New Amsterdam in 1867. It has now been renamed as the National Psychiatric Hospital; however, despite the change of name, stigma continues to be attached to this institution. Until 2022, Guyana’s primary piece of legislation to guide mental health service delivery was the Mental Health Ordinances of 1930. More recent initiatives have been established in Guyana to provide mental health care, which include community health centers that provide mental health services, collaborative work between public health, and other initiatives [31].

Despite the recent developments which have sought to extend provision in mental health care, as part of the National Suicide Prevention Plan, the remnants of the colonial past also persist in this area of social life. The following has been noted by those working in this field:

*Many people do not reach out for mental health support because they are afraid they will be locked up for an extended period. Eighty-five percent of patients sent for treatment spend more than five years in long-term care in a psychiatric hospital, and there are no laws that protect the rights of patients to refuse services or leave the hospital on their own accord. Many people, then, might keep struggles with depression or suicidal thoughts quiet for fear of being locked away at the National Psychiatric Hospital, which many still refer to as the “Berbice Madhouse”.*

[21]

While the recent Mental Health Protection and Promotion Bill (2022), which calls for “a mental health promotion policy [to be] prepared to address the determinants of mental health, promote wellness, prevent suicides and substance abuse” [32], is very welcome and offers an opportunity for mental illness to be examined within a sociogenic lens, the decision-makers within the legislation have not sufficiently sought to include those who have expertise in doing community-based work in this area. The Mental Health Bill of 2022 consists of vital indicators to progress from the service provision of the previous Mental Health Ordinances Act of 1930 [33]. However, what is crucial in fulfilling the provisions made in this Bill and providing access to culturally appropriate care is the prevailing attitudes about mental illness, along with the implementation and monitoring of the Bill. Effective monitoring and implementation are critical since a country report [34] has highlighted that a gap or breakdown exists in implementing similar legislation, such as the Prevention of Discrimination Act of 1997.

## 5. Obeah and Traditional Forms of Addressing Psychiatric Distress

Guyana is a strongly religious nation, and the three religions of Hinduism, Islam, and Christianity are very widely practiced. The belief in a God or higher power serves many purposes, including blessings, guidance, protection from harm, and intervention in mental and emotional distress. Many people seek and find solace through prayers, reading scriptures or consultation with religious leaders. However, there is another level to this which we see as important to raise within this discussion, and this involves the belief that forms of psychological distress are a product of “spiritual attacks” or demonic possession [35,36,37]. Such interpretations are grounded in the belief in the supernatural known primarily in Guyana as “Obeah” but which are also called black magic or voodoo. While the practice of Obeah goes against the principles of official religion, James et al. [38] point to the way that for many people, and particularly the poorest and most marginalized, this conception of supernatural causation is the lens through which they understand and seek support around mental illness. These belief systems pre-date the colonial period in which this was the main source of healing available. While colonial rule introduced western biomedical practices, these facilities were essentially set up to meet the needs of the white population; it was also the case that during colonialism, the practice of Obeah was considered evil and pagan and was made illegal, with severe punishment if slaves were found to be involved in its practice [39,40].

According to Bilby and Handler [41] (p. 154), the belief and practice of Obeah are grounded in two main characteristics:

(1) Its practice involved the manipulation and control of supernatural forces, usually using material objects and recitation of spells;
(2) It was primarily concerned with divination (e.g., foretelling, finding lost or stolen goods, ascertaining the cause of illness), healing and bringing good fortune, and protection from harm, although it was sometimes used malevolently to harm others.

There is no published research on the extent of these practices in Guyana, but research undertaken in Jamaica offers comparable evidence. Arthur and Whitley [42] have undertaken research on people’s beliefs regarding the causation of mental illness using focus groups. While their respondents noted drug-related causes, biological causes (including “blood”), and social factors such as stress and insecurity, their work also demonstrates widespread belief in supernatural causation. James and Peltzer’s [43] study concerns attitudes of patients’ in Jamaica’s psychiatric hospitals to mental ill health causation, and this research demonstrated that over a third believed the overall cause of their mental illness was as a result of supernatural factors. These patients also believed the diagnosis provided by the traditional healer was more reliable than that of a medically trained professional. These and other studies [44,45] point to widespread distrust in the biomedical mental health model alongside belief in supernatural causation of mental illness, often linked to witchcraft and demonic possession. Seeking help from medical professionals in this context was often the last course of action after traditional healers were unable to provide healing [46].

There are a number of dimensions to this phenomenon. First, “madness” continues to be understood throughout the Caribbean as a deep source of shame. A diagnosis of a mental illness significantly damages people’s social status, leading to othering and exclusion from society [47,48]. Arthur et al.’s [39] study “Mad, Sick, Head Nuh Good: Mental Illness Stigma in Jamaican Communities”, points to the way in which stereotypical views of mental illness create significant stigmatization for people diagnosed, who come to be seen as objects of fear in the wider community. Negative representations of people with mental illness abound in the media, where they are often presented as engaged in criminal activities, are shown to be excluded from the communities they have grown up in, and, in some instances, are physically attacked [48,49,50]. It is not surprising, as Ellis [51] notes, that this stigma contributes to widespread denial when people begin to experience mental illness, and this itself contributes to already extensive abuse of alcohol and substances [52,53]. This body of research points to the mutually compounding nature of the problem, where denial can worsen the initial problem, leading to people suffering in silence, self-medicating with alcohol or drugs, or seeking alternative sources for help such as Obeah.

It is in this context that we need to understand why the ongoing utilization of traditional medicine and reliance on the supernatural to treat sicknesses continues to be prevalent in Guyanese society despite attempts to develop the use of medication, psychotherapy, and other contemporary resources. According to Snow [40], traditional medicines are seen to offer solace and comfort to individuals and can give people who use them a sense that they have control over what is happening. Waldron [54] has also argued that western biomedical domination has suppressed the validity and usefulness of traditional forms of healing, which form part of the African people’s identity. However, reliance on the supernatural and traditional forms of healing fails to do anything to challenge the structural inequalities which we argue are at the root of the severe levels of psychiatric distress seen in Guyana [55,56,57]. Sutherland’s study of traditional healing and spirituality amongst women on the Caribbean Island of Grenada [23] offers insight in this respect. She argues that, faced with ongoing violence towards women, traditional healing is one of the few resources women have:

*Traditional healing, with its focus on the body, is a culturally appropriate approach for enabling these women to go through the corrective process of reconstituting the self by immersing themselves in and symbolically acting-out the trauma that appears to be contained within the body, a particularized trauma that seems to defy verbal representation.*

[23]

At the same time, Sutherland’s findings also point to the way in which these forms of spirituality can be like “opiates”, analogous the way Karl Marx described the role religion played in the lives of the oppressed (the full quote from Marx’s *A Critique of Hegel’s Philosophy of Right* (1844) is as follows: “Religious suffering is at one time the expression of real suffering and a protest against real suffering. Religion is the sigh of an oppressed creature, the heart of a heartless world and the soul of soulless conditions. It is the opium of the people. The abolition of religion as the illusory happiness of the people is the demand for their real happiness” [58] (p. 244)). When means of securing justice through the state and the law are almost entirely absent, and given the structural barriers these women experience, they have little else to turn to apart from their traditions. Sutherland quotes Siegfried Nadel’s argument to the effect that

*…Witchcraft beliefs enable a society to go on functioning in a given manner, fraught with conflicts and contradictions which the society is helpless to resolve; the witchcraft beliefs absolve the society from a task apparently too difficult for it, namely, some radical readjustment.*

[59] (p. 45)

Alongside this, it is important to note that as Khoury et al. point out, it is the most marginalized, poor, and illiterate victims of violence who are also the most likely to use traditional healers [45] (p. 528). Where feelings of powerlessness exist, people gravitate to non-threatening spaces and entities that offer reassurance. However, in cases of severe mental distress, genuinely therapeutic interventions are needed, which offer more than reassurance. Nortje et al.’s research on the use of traditional healers in Southern Africa points the way in which these can provide an effective psychosocial intervention which “might help to relieve distress and improve mild symptoms in common mental disorders such as depression and anxiety. However, little evidence exists to suggest that they change the course of severe mental illnesses such as bipolar and psychotic disorders” [60] (p. 1). (There is an important debate in the international literature on decolonizing mental health, which considers the importance of incorporating traditional healers as part of modern mental health provisions in countries such as Guyana, for example, as part of a referral pathway. Dudgeon and Bray argue in the context of mental health issues amongst Indigenous people in Australia that “traditional healing is widely believed to be the most efficacious way to assist distressed First Nations individuals due to the inherent potency of these traditions achieved through long pre-contact histories of therapeutic refinement” [61] (p. 98) We would see the situation with Obeah as more problematic, and it is important to note that ritualist practices in Guyana are not simply benign and can include beating, walking on objects, drinking potentially harmful potions, and even engaging in illegal vigilante justice [16,62]. Our general argument here is that a sociogenic approach is needed which transcends the individualist focus of both Obeah and biomedical provision.)While the belief in the supernatural and traditional forms of healing needs to be acknowledged and appreciated within their context, our fundamental argument concerns the need for greater acknowledgement of the sociogenic understanding of mental illness.

## 6. Mental Health Social Work in Guyana: Building a Different Future

In the context described so far, we argue that a nuanced understanding of suicide and mental illness in Guyana is critical. Within Guyana’s “broken heritage”, the very understanding of the psychiatric distress which so marks the lives of the people is, to a large degree, polarized between biomedical interventions and the supernatural. While biomedical provision can play an important role in addressing and alleviating psychic distress, the medical diagnoses surround those diagnosed with stigma, and within the forms of provision available, there is a lack of rights-based treatment which would allow the users of these services a voice in terms of the direction of the support they receive, a factor which reinforces the stigmatized nature of this provision. However, at a deeper level, there is a fundamental problem with the way in which the biomedical approach locates the causation of mental illness at the individual level, failing to engage with the way in which so much psychic distress in Guyana is socially produced and directly connected to a range of forms of violence which are commonly experienced in everyday life. It is in such a situation that some people turn to Obeah and other spiritual practices, and while this is understandable, like traditional biomedical models, these approaches equally fail to address the sociogenic nature of psychic distress.

We have argued that the problem of the “broken heritage” shapes contemporary psychiatric distress in Guyana, as social identities and institutions continue to reflect the legacy of colonialism. Acknowledging that the suffering of individuals results from the trauma of the colonial legacy requires us to consider new models of mental health intervention which consciously problematize colonialism and point to its ongoing legacy in Guyana. We see a social work model of social rights and empowerment as uniquely positioned to deliver such a model, which would incorporate users’ lived experiences as well as being based on a human rights framework for people affected by mental ill health. Orr and Jain, writing on the context of the WHO Global Mental Health agenda, argue that “social work, with its underpinning theory base constituted in the intersection of the psychological, social, political and economic contexts, is well placed to contribute” [63] (p. 574), and this point is one which we see as very pertinent in the Guyanese context.

The form of social work and mental health support offered and the ways in which these are undertaken are crucial here. While social work is present in contemporary Guyana, it significantly lacks visibility as a profession. In order to address this, there is a need for social workers to acquire a license which allows them to become a recognized profession (discussion is already beginning on this in Guyana; see https://demerarawaves.com/2021/03/21/social-workers-need-legal-regulation-more-involvement-in-policy-making/((accessed on 1 August 2023)). This is important as a means of facilitating ways for the profession to become more autonomous from the government, both in developing new directions in social policy and in being able to question and challenge government policy. The integrative and sociogenic approach we are calling for here means that social workers need to be able to talk openly about the kinds of issues people are facing, not just as personal problems but as social issues which are constituted within law, government policy, and state institutions, as well as wider sets of power relations. The level of suicide in Guyana is a profound manifestation of Fanon’s understanding that the psychic distress of the individual results from the internalization of social tensions and, in particular, from the way that “violence is a normalized experience, an everyday encounter for Guyanese people” [22] (p. 22). In this sense, the problem of suicide in Guyana reflects the state of the country as a whole, and social workers need to be able to act as advocates and agents of change in order to address this. Jessica Horn offers a useful model from mental health social work in Africa, where she notes that “For people born into war, economic marginalization, and the discriminatory norms of racism, sexism, homophobia, and xenophobia, the world itself is the stressor. This means, in turn, that activism is necessary to change the structural roots of distress, while individual or group therapies are engaged to build the capacity to manage or engage the effects of these structural stressors in everyday life” [64] (p. 90). Social work is one of the few professions which is able to offer this combination of advocacy work and more directly therapeutic work with individuals, groups, and communities, and this is very much the way in which we would argue that social work should develop in Guyana.

Alongside the development of a more autonomous professional identity, it is also important to think about social work’s role within mental health provision itself. Inherent within the sociogenic approach is an attempt to shift the focus from the “mad” person as an object of fear and loathing toward an understanding of the social tensions which create the conditions for mental breakdown. In situations where this takes place, social work plays an important role in ensuring that treatment is compassionate, humane, and based in an acknowledgment of people’s rights. The models of provision developed in Jamaica by the Caribbean Institute of Mental Health and Substance Abuse, established in collaboration with the University of the West Indies, represent the kind of innovative approach that needs to be understood and appreciated far more widely [65]. The approach developed here is community-based and culturally localized, and this offers an important model for Guyana [65]. Social work plays an important role in championing these new forms of provision, which offer an important alternative to the stigma of psychiatric hospitals. However, as well as calling for new forms of community-based provision, social work can be involved in assisting the transformation of existing forms of provision. It is important to remember that Fanon’s work at the Blida-Joinville Psychiatric hospital is an example of the way in which services which were set up along highly oppressive and hierarchical guidelines can be reconfigured on a new basis, creating new models of recovery [14]. Even within hierarchical and oppressive institutions, Fanon found ways in which to engage these people as active agents in their own recovery. Social work education has an important role to play in teaching new generations of practitioners about this important history and the way it links together the political critique of colonialism and its contemporary aftermath with the process of developing rights based and emancipatory mental health provision. While social work can play an important role in this work, social workers also need to be working collaboratively with other health-based professions, working inside and outside traditional biomedical forms of provision. The development of these initiatives can enable social workers to create a unique identity and approach in the mental health sector, based on a philosophy of equality and “open dialogue” (while there is insufficient space to talk about how the model of “open dialogue” in mental health could work in Guyana, there is evidence which points to the this elsewhere; see McKenzie-Brooke [66]; these approaches draw on the work of the Brazilian educator Paulo Freire, who is well known throughout Latin America and the Caribbean), an approach which is based on giving patients and service users a voice in their own treatment.

## 7. Conclusions

We began our discussion with reference to the Guyanese author Wilson Harris, suggesting that his concept of a “broken heritage” offers an important and useful means of conceptualizing the way in which the dynamics of the colonial period shape identities in the present. Within this discussion, we have argued that the high rates of suicide which persist in Guyana need to be understood sociogenically, and that we need to situate these mental health problems generally in a wider context of the violence and structural barriers which are prevalent in the social life of contemporary Guyana, and which reflect that colonial history. Samuel Durrant has argued that for Wilson Harris, Guyana’s history cannot offer “a sense of community in which individuals are bound together by a common cultural inheritance but [rather] by a collective experience of loss and by a shared sense of responsibility for this loss” [67]. The reconciliation which needs to come from this broken heritage needs to start with an understanding of the profoundly psychically damaging nature of colonial social relations, and why it is so important to problematize these. Social work is in a unique position to contribute to this different future by imagining and building new forms of mental health support and provision which name the colonial past while at the same time create space for new constructions of identity and less oppressive social relations in Guyana. We hope that the issues identified in this paper can contribute to further research and discussion about the directions of mental health practice and interventions in Guyana.

## Data Availability

All data supporting arguments made in this discussion is referenced within the article.
